# Experiences of Physical Therapists and Professional Leaders With Implementing a Toolkit to Advance Walking Assessment Poststroke: A Realist Evaluation

**DOI:** 10.1093/ptj/pzab232

**Published:** 2021-10-04

**Authors:** Nancy M Salbach, Alison McDonald, Marilyn MacKay-Lyons, Beverly Bulmer, Jo-Anne Howe, Mark T Bayley, Sara McEwen, Michelle Nelson, Patricia Solomon

**Affiliations:** 1 Department of Physical Therapy, University of Toronto, KITE-Toronto Rehabilitation Institute, University Health Network, Toronto, Ontario, Canada; 2 Nova Scotia Health Authority, Halifax, Nova Scotia, Canada; 3 School of Physiotherapy, Dalhousie University, Halifax, Nova Scotia, Canada; 4 Unity Health Toronto, Toronto, Ontario, Canada; 5 Department of Physical Therapy, University of Toronto, Toronto Rehabilitation Institute, University Health Network, Toronto, Ontario, Canada; 6 KITE-Toronto Rehabilitation Institute, University Health Network, Department of Medicine, University of Toronto, Toronto, Ontario, Canada; 7 Selkirk College, Castlegar, British Columbia, Canada; 8 Lunenfeld-Tanenbaum Research Institute, Sinai Health System, Institute of Health Policy, Management and Evaluation, Dalla Lana School of Public Health, University of Toronto, Toronto, Ontario, Canada; 9 School of Rehabilitation Science, McMaster University, Hamilton, Ontario, Canada

**Keywords:** Guidelines, Knowledge Translation, Standardized Assessment, Stroke, Toolkit, Walking

## Abstract

**Objective:**

The iWalk study showed significant increase in use of the 10-Meter Walk Test (10MWT) and 6-Minute Walk Test (6MWT) poststroke following provision of a toolkit. This paper examined the influence of contextual circumstances on use of the toolkit and implementation strategy across acute care and inpatient and outpatient rehabilitation settings.

**Methods:**

A theory-based toolkit and implementation strategy was designed to support guideline recommendations to use standardized tools for evaluation of walking, education, and goal-setting poststroke. The toolkit comprised a mobile app, video, and educational guide outlining instructions for 3 learning sessions. After completing learning sessions, 33 physical therapists and 7 professional leaders participated in focus groups or interviews. As part of a realist evaluation, the study compared and synthesized site-specific context-mechanism-outcome descriptions across sites to refine an initial theory of how the toolkit would influence practice.

**Results:**

Analysis revealed 3 context-mechanism-outcomes: (1) No onsite facilitator? No practice change in acute care: Without an onsite facilitator, participants lacked authority to facilitate and coordinate the implementation strategy; (2) Onsite facilitation fostered integration of select practices in acute care: When onsite facilitation occurred in acute care, walk test administration and use of reference values for patient education were adopted variably with high functioning patients; (3) Onsite facilitation fostered integration of most practices in rehabilitation settings: When onsite facilitation occurred, many participants incorporated 1 or both tests to evaluate and monitor walking capacity, and reference values were applied for inpatient and outpatient education and goal setting. Participants preferentially implemented the 10MWT over the 6MWT because set-up and administration were easier and a greater proportion of patients could walk 10 m.

**Conclusion:**

Findings underscore contextual factors and activities essential to eliciting change in assessment practice in stroke rehabilitation across care settings.

**Impact:**

This study shows that to foster recommended walking assessment practices, an onsite facilitator should be present to enable learning sessions and toolkit use.

## Introduction

Stroke rehabilitation guidelines provide recommendations to guide diverse aspects of physical therapist practice.^1–4^ Toolkits are a potentially low-cost strategy to support widespread implementation of recommendations for assessment and treatment. Toolkits are considered complex interventions because they comprise multiple components and may target various behaviors of people in different roles in a complex clinical environment.^5^ During development of complex interventions, it is recommended that both quantitative and qualitative analysis be used to understand contextual characteristics of practice settings and active mechanisms of the individual toolkit components that influence implementation.^6^

We developed a theory-based toolkit (iWalk toolkit) to guide physical therapists in using the 10-Meter Walk Test^7^ (10MWT) and 6-Minute Walk Test^8^ (6MWT) for test administration and interpretation, patient education of performance, goal setting, and treatment selection poststroke.^9^ These measures of walking are highly recommended across stroke care settings^1^^,^^4^^,^^10^^,^^11^; however, clinical application has been inconsistent.^12^^,^^13^ The toolkit consisted of a mobile app,^14^ educational video,^15^ and an educational guide^16^ that described an implementation strategy involving 3 group-learning sessions organized by a local physical therapist or practice leader. Table 1 outlines the toolkit components and implementation strategy. The implicit theory underpinning the toolkit was that reviewing and discussing information in the guide, video, and app and practicing the suggested activities would enhance understanding of the implications and potential benefits of the walk tests and increase knowledge, skill, and self-efficacy to perform the recommended practices and, ultimately, clinical implementation.^9^

**Table 1:** iWalk Toolkit Components and Implementation Strategy*^a^*

**Intervention Component**	**Description**
**iWalk Guide Modules**	
Module 1: Introduction	Top 10 reasons to use iWalk and related Canadian guideline recommendations*^b^*
Module 2: Performing the tests	Walk test protocols and pictorial instructions for people with aphasia
Module 3: Interpreting test performance	How to interpret test performance using normative values, crosswalk speeds, community distances, and minimal detectable change values using patient examples
Module 4: Educating and setting goals	How to educate patients about test performance and goal setting, using patient examples
Module 5: Selecting treatments	Treatment approaches known to improve walking speed and distance measured using walk tests
Module 6: Evaluating practice using audit and feedback	How to use audit and feedback or group discussion to reflect on practice change Potential implementation challenges and how to overcome them
Module 7: Putting it all together with case scenarios	Inpatient and outpatient case scenarios and learning sessions to be completed
Module 8: Appendix	Instructions and agendas for 3 learning sessions Equipment and space requirements checklist Printable walk test protocols and aphasia-friendly instructions Printable data collection and goal setting forms Printable 1-page summary of reference values Printable Rating of Perceived Exertion scale Patient education tool for documenting test results and goals
**iWalk Video** (duration: 11 min)	Demonstration of a physical therapist administering each walk test with a person poststroke Educational content related to walk test protocols, interpretation, and documentation in the health record
**iWalkAssess App for iOS and Android**	Outlined stroke-specific protocols Written and audio-recorded instructions Timing tools (10MWT stopwatch; 6MWT countdown timer, length counter by swiping the screen, display of standard instruction each minute with vibration alert to administer tests, with the option to enter test results manually), and computation of test results Automated algorithms comparing test results with reference values (ie, age- and sex-specific norms, community ambulation speeds and distances, and minimal detectable change values) Reference section with definitions and references
**Implementation strategy** ^***b***^	
Learning session 1	Review Module 1, view video, practice tests, discuss implementation Presession: review Module 2 Homework: use tests with 1 patient before Session 2, and document test results in health record
Learning session 2	Enter data into app from a case scenario, interpret performance, complete forms, review Module 5 Presession: review Module 3 Homework: use tests with 2 patients before Session 3, and document test results and interpretations in health record
Learning session 3	Role-play goal setting based on a case scenario, discuss feasibility of implementing audit and feedback Presession: review Module 4 Homework: use tests with 1 patient, document test results and interpretations in health record, educate the patient, and set goals using the patient education tool

^*a*^6MWT = 6-Minute Walk Test; 10MWT = 10-Meter Walk Test.

^*b*^Sites were asked to identify a facilitator responsible for setting up walkways and organizing and facilitating 3 learning sessions. (The term “facilitator” refers to an individual who provides the support needed to help clinicians to successfully implement a new evidence-based practice.^40^^,^^41^) Sites were given access to an expert physical therapist with 24 years of experience treating people poststroke who could answer questions by email or phone throughout the study.

**Figure:**
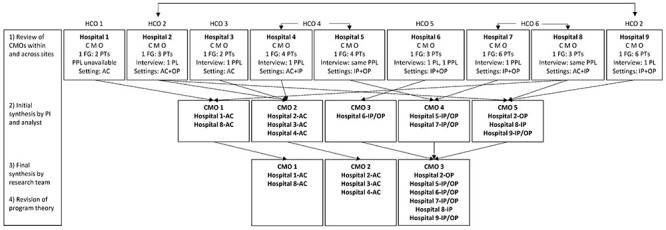
Realist analysis and data sources. AC = acute care; C = context; FG = focus group; HCO = healthcare organization; IP = inpatient; M = mechanism; O = outcome; OP = outpatient; PL = professional leader; PPL = professional practice leader; PT = physical therapist.

Previously, we conducted a quantitative evaluation of the use of the toolkit among physical therapists providing acute care and inpatient or outpatient stroke rehabilitation at 9 hospitals employing professional practice leaders (PPLs) or professional leaders (PLs).^9^ PPLs and PLs are health care professionals responsible for advancing evidence-based practice and ensuring attainment of professional practice standards of 1 or more professional groups within a hospital organization.^17^ PPLs (but not PLs) treat patients. We found that physical therapists were significantly more likely to administer the walk tests post intervention compared with pre-intervention, with implementation of the 10MWT improving to a greater extent than the 6MWT. The magnitude of improvement was lowest in acute care and highest in outpatient rehabilitation settings.^18^

Although these quantitative findings suggest some benefit of toolkit provision, they provide little guidance on how sites might overcome challenges and maximize implementation success to improve outcomes. In particular, the role of the PPL or PL in facilitating toolkit implementation is unclear. Realist evaluation^19^ is ideally suited to understanding “what works, for whom, under what circumstances, and how” in complex clinical settings.^20^ Realist evaluations focus on describing the variable ways in which contextual factors and mechanisms interact to produce outcomes (context-mechanism-outcome [CMO] configurations) during a practice change initiative. For example, a hospital might not have sufficient space to conduct a walk test near the patient treatment area (contextual factor). Thus, despite gaining knowledge and skill from reviewing the toolkit and practicing the walk test (mechanism), therapists may seldom administer the walk test in clinical practice (outcome). CMO configurations are transferable to similar practice settings and can inform future practice change endeavors. Thus, to complement our quantitative evaluation, we conducted a realist evaluation, which is the focus of this paper. The overall purpose was to examine how the contextual circumstances of acute care and of inpatient and outpatient rehabilitation practice settings influenced participants’ engagement with the toolkit and implementation strategy to effect practice change. This type of inquiry is essential to refine program theory and advance implementation science in the rehabilitation setting.

## Methods

### Study Design

We followed the approach to conducting a realist evaluation proposed by Pawson and Tilley^19^ and used RAMSES,^20^ SRQR,^21^ and TIDiER^22^ checklists to guide reporting. The study was designed and conducted with end-users of the toolkit on the research team. The research ethics board at the University of Toronto (Protocol 31232) and each participating hospital approved the study protocol. Participants provided written informed consent.

### Eligibility and Recruitment

Hospitals providing acute care or inpatient or outpatient rehabilitation services for people poststroke and physical therapists providing walking rehabilitation to ≥10 patients poststroke per year were considered eligible. Using convenience sampling, we identified 6 general and 3 rehabilitation hospitals in 2 urban centers (Toronto, Ontario; Halifax, Nova Scotia) and emailed PPLs/PLs an invitation to participate. All PPLs/PLs agreed and provided a letter of support for our grant application. After obtaining funding, PPLs/PLs helped schedule recruitment sessions at each site at which we invited physical therapists and PPLs/PLs to participate.

### Intervention

In September 2015, we mailed to each site a package containing printed copies of the iWalk guide; Android smartphones for 22 physical therapists who did not own a smartphone or were unwilling to use their personal smartphone; and lanyards for smartphones to enable safe, hands-free administration of the walk tests. We also emailed participants an electronic copy of the iWalk guide and a private YouTube link to the iWalk video. Sites were asked to identify a facilitator (eg, PPL/PL, physical therapist, manager) responsible for setting up walkways and organizing and facilitating 3 learning sessions within a 5-month period.

### Data Collection

On completion of learning sessions, a Toronto-based female interviewer with a master’s degree and 2 years of qualitative research experience, who was not known to participants, conducted 1-hour focus groups with physical therapists in-person at hospitals in Ontario and 1-hour focus groups with physical therapists and a site facilitator by phone at hospitals in Nova Scotia. PLs and PPLs were interviewed by phone. Three semi-structured guides with probing questions were developed for physical therapists, PPLs, and PLs (for sample questions, see Suppl. File).

The interviewer shared participants’ responses, allowing them to reflect and build on past interviews or focus groups, a form of transactional validity in which member checking is part of the data collection process.^23^ Focus groups and interviews were audio-recorded and transcribed verbatim. Data on sociodemographic and practice characteristics were collected using an online questionnaire, described in detail elsewhere,^9^ administered using Fluid Survey v3.0 in August 2015 prior to sending the toolkit.

### Data Analysis

A directed qualitative content analysis was performed.^24^ Two authors (N.M.S. and P.S.) developed a codebook with definitions for context, mechanism, and outcome (Tab. 2). The 14 domains of the Theoretical Domains Framework^25^ (TDF) were used as codes to describe contextual characteristics. The TDF domains (eg, knowledge, skills, beliefs about capabilities, environmental context, and resources) were derived from behavior change theories and represent a range of barriers and enablers that may influence practice change interventions. One author (N.M.S.) and a research assistant independently coded 4 transcripts, then met to discuss the results, resolve differences, and revise the codebook. The research assistant used NVivo10 software to code data, including transcripts and reflective notes, from 1 hospital at a time. Data from PPL/PL interview(s) and focus group(s) with physical therapists within each site were compared to develop a CMO summary with supporting quotes for each site. Author N.M.S. and the research assistant compared and contrasted the CMO summaries within and across practice settings to produce 5 CMOs. Six research team members (4 from participating hospitals), including researchers, physical therapists, PPLs, educators, and hospital administrators, met to review, discuss, and further synthesize the CMO summaries, producing 3 CMOs. The Figure describes the process of the realist analysis as well as the data sources and practice settings of each site.

**Table 2:** Definitions of Context, Mechanism, and Outcome in the Realist Analysis^a^

**Primary Codes**	**Definition**	**Subcodes**
Context	Preexisting features of setting or microsystem (eg, characteristics of physical therapists, patients, practice leaders, policy, group functioning, physical environment). into which interventions are introduced that are relevant to the operation of the mechanism or that impact the mechanisms (eg, position held by a facilitator, and facilitator’s beliefs, would be relevant to the mechanism).^42^	14 domains of TDF For the TDF domain environmental context and resources, 3 subcodes for patients, space, and time were developed
Mechanism	The process(es) through which participants interpret and act on the interventions (ie, iWalk resources), including features of the resources that affect, or do not affect, change.	Intervention components (ie, guide, app, video, walkway set-up, learning sessions, clinical expert)
Outcome	Intended consequences (ie, implementation of recommended practices) and unintended consequences resulting from the occurrence of different mechanisms in different contexts. Also includes deciphering the reasons behind why the outcomes occur.	Each walk test, walk test not specified, treatment selection, and unintended outcomes

^*a*^TDF **=** theoretical domains framework.

Credibility and rigor of the data analysis were ensured through active probing for detail during data collection, collection and analysis of reflective notes, sharing experiences across sites, verifying data accuracy by comparing transcripts to audio-recordings, triangulating data from focus group and interview participants at the same site, and discussing results with research team members working in similar settings.

### Role of Funding Source

Funders played no role in the design, conduct, or reporting of this study.

## Results

Nine focus groups with 33 therapists providing acute (39%), inpatient (39%), and outpatient (21%) rehabilitation and 7 interviews with 4 PPLs and 3 PLs were conducted. All PPLs and PLs were female physical therapists, and 2 PPLs oversaw practice at 2 hospitals. Table 3 presents characteristics of participating physical therapists.

**Table 3:** Personal and Practice Characteristics of Participating Physical Therapists (n = 33)^a^

**Characteristic**	**n (%)**
Age, y	
20–29	3 (9%)
30–39	19 (58%)
40–49	6 (18%)
50+	5 (15%)
Female sex	29 (88%)
Highest degree	
Certificate/diploma	1 (3%)
Bachelor’s	20 (61%)
Entry-level master’s	10 (30%)
Applied/research master’s	2 (6%)
Clinical experience, y	
1–5	5 (15%)
6–10	8 (24%)
11–15	11 (33%)
16–20	2 (6%)
>20	7 (21%)
Poststroke patients, no./wk	
<2	5 (15%)
2–5	14 (42%)
6–10	8 (24%)
>10	6 (18%)
Primary type of care delivered	
Acute, inpatient	13 (39%)
Rehabilitation, inpatient	13 (39%)
Outpatient	7 (21%)
Learned to administer 10MWT during professional program	8 (24%)
Learned to administer 6MWT during professional program	24 (73%)

^*a*^6MWT = 6-Minute Walk Test; 10MWT = 10-Meter Walk Test.

Implementation fidelity and participant engagement with the intervention have been previously reported.^9^ Briefly, the percentage of physical therapists that reviewed each iWalk guide module ranged from 62% to 97%. Eighty-three percent reported viewing the video, and 75% used the app for at least 1 month.

We identified 2 CMOs in the acute care setting and 1 CMO in settings with inpatient and/or outpatient rehabilitation. Table 4 summarizes the contextual factors, classified within TDF domains, mechanism, and outcome for each CMO, with supporting quotes.

**Table 4:** CMO Results Matrix^a^

**Contextual Variables,** ^***b***^ **Mechanism of Action, Outcome, Quotes**	**CMO1: No Onsite Facilitator? No Practice Change in Acute Care** **(2 Hospitals)**	**CMO2: Onsite Facilitation Fostered Integration of Select Practices in Acute Care** **(3 Hospitals)**	**CMO3: Onsite Facilitation Fostered Integration of Most Practices in Inpatient and Outpatient Rehabilitation** **(6 Hospitals)**
Context			
Environmental context/resources	• No. of PT-participants at each hospital: 2, 1	• No. of PT-participants at each hospital: 2, 2, 2	• No. of PT-participants at each hospital: IP: 2, 3, 3, 5, 6; OP: 1, 1, 1, 1, 1
Human resources	• PPL unavailable or offsite	• PPL/PL onsite or offsite	• PPL/PL onsite in 5 of 6 hospitals
Space	• Difficulty finding a safe 30-m hallway space for 6MWT walkway close to treatment area • Issues included hallway curving, walkway passing through fire doors, and intersecting hallways causing safety concerns • High level of clutter (eg, equipment, patients on stretchers) and traffic in hallways • Health care professionals and visitors often walking in hallways and exiting patient rooms, which could lead to a collision or interrupt the walk test and distract the patient, requiring the walk test to be redone	• Same as CMO1	• Difficulty finding safe 30-m hallway space for 6MWT walkway close to treatment area • Hospitals with IP and OP settings needed to set up walkways in at least 2 locations
Time	• Initial and follow-up evaluations 20–30 min • Evaluations brief due to low patient tolerance • Diagnostic tests, such as MRIs and X-rays, take precedence over physical therapy sessions • Completing AlphaFIM^41^ is mandatory to refer patients to rehabilitation in Ontario	• Initial evaluations 10–60 min • Otherwise, same as CMO1	• Initial evaluations 30–60 min for IP and OP settings • IP therapists have flexibility to complete evaluations over multiple sessions
Patients	• Majority of patients cannot walk or have low levels of walking ability • Patients often fatigued because of effects of stroke combined with extensive medical testing	• Same as CMO1	• IP units often had a high-intensity program (therapy 6×/wk) and slow stream program (therapy 3×/wk) • Trend towards admitting people with severe sensorimotor deficits to IP and referring people with a mild stroke to OP services • IPs often non-ambulatory and OPs typically ambulatory on admission
Knowledge and skills	• PTs not using tests in clinical practice	• PTs at 2 sites had experience using walk tests from a previous study but were not using them in clinical practice	• PTs at select sites were already using 1 or both walk tests in clinical practice
Beliefs about consequences	• Walk tests not viewed as beneficial to clinical practice; some believed 6MWT performance in a patient unable to walk 6 min without stopping is not valid	• Leaders and majority of PTs had positive attitudes towards using walk tests in high functioning patients	• Leaders and majority of PTs had positive attitudes towards using walk tests
Goals	• Major focus of assessment was discharge planning • Length of stay benchmarks in Ontario were 3 d for TIA or mild stroke, 5 d for ischemic stroke, and 7 d for hemorrhagic stroke • Primary therapy goals: improve basic mobility (ie bed mobility, transfers, and standing balance) and safe use of prescribed gait aids	• Same as CMO1	• Primary therapy goals: balance and mobility (IP); advanced balance and mobility (OP)
Mechanism	• PTs did not set up permanent walkways or complete learning session activities as prescribed due to inadequate leadership and facilitation, and perceived limitation in professional role • Lack of local leadership and perceived irrelevance of walk tests to patients in acute care setting resulted in negative attitudes toward study involvement	• PPL/PL provided leadership and facilitation • PTs at all sites marked walkways, completed learning session activities, including practicing walk tests, and used reference values with patients • As a result, PTs observed a benefit to patients that fostered positive beliefs in the consequences of administering the walk tests • Due to limited time, select PTs prioritized measures (eg, Berg Balance Scale^26^) or observational gait assessment as these methods provided more valuable information than walk tests	• With facilitation by PPLs and PLs, PTs engaged with implementation strategies: they completed learning sessions, set up walkways, practiced tests, and used reference values (ie age/sex norms, crosswalk speed, community/household ambulator classification) with patients • PTs were more successful implementing 10MWT than 6MWT because it was easier to find space for walkway near treatment area, took less time to complete, and a greater proportion of patients could complete test
Outcome	• No intention to implement recommended practices despite gains in knowledge and skill	• Integration or intentions to implement select recommended practices in high functioning patients who can tolerate walking 6 min without rest	• Majority of PTs described applying recommended practices in IP and OP rehabilitation settings
Quotes from PPLs and PLs	“Most of the therapists that worked in [the hospital where PPL was onsite] were in the rehab setting. We were all on the same floor. We set up an area that we already had a measured 10 meter walk. In [the hospital where PPL was offsite], they did have more challenges...And it was them that set it up. So I wasn’t involved in setting it up. (PPL, hospital 8)	“It’s important that the [facilitator] does have a clinical component to their role…treating patients. Because then [therapists] can relate and say: you know what I’m going through, you’re working with the [same] types of patients and schedules that I’m working with. And if you can do it, I can do it.” (PPL, hospital 4)	“As a participant, I had an open view of what you were asking us to do and about following some of the recommendations. Trying to facilitate the discussion with other physios who disagreed with me and maybe thought some things were not necessary...was really difficult as it ran contrary to my beliefs.” (PPL, hospital 6)
Quotes from physical therapists	“To make it feasible [the walkway] needs to be in the space where you’re already going to do your regular assessment…so that it’s just an easy thing to do. And to get manager support for having a permanent location for the course, and assistance in setting that up.” (hospital 1) “I found it difficult to organize ourselves as physios in different units to do the learning sessions together. There almost needs to be a champion or 1 person on site that was in charge of coordinating us and getting us together because we are all on different committees and have different clinical commitments. And then we didn’t do the steps on each other. We were just behind the whole time.” (hospital 1) “Sometimes I’m reluctant to talk about performance or how well they’re going to do because they’re so soon after their stroke. I don’t want to say you’re going to be amazing and this will be your goal, and then they won’t achieve that.” (hospital 1)	“I found that the [walk tests] that I did, it was more just to inform the patient. But then they were gone. And so there was no carry forward with the information that I had. And I kind of hope…because it made it into my documentation…whatever therapist used them in the future, they might be able to utilize that as a baseline.” (hospital 2) “I don’t as a routine practice initiate something that’s going to be discouraging to my patients... Especially when you’re dealing with somebody who’s had a rapid change in their function and they’re in a grieving process. You need to build their confidence, not detract from it. I generally wouldn’t do something like a 6 minute test until somebody actually has the endurance to do it...with the appropriate amount of assist. So that narrows it down from an acute care point of view. I’m more likely to use a 10 meter [walk test] to get a gauge of their general gait speed...And I usually [use the] NuStep to work on their endurance in a safe way rather than doing the 6MWT.” (hospital 4) “I definitely feel like the patients that we did do [the 6MWT] with got something out of it. So if we have the time and space to do it…I certainly would be happy and willing to adopt this into my everyday practice if we had the appropriate patient who’s with us long enough.” (hospital 3)	“I just had a patient discharged this week …his 6-minute walk value was still only 50% of norm for his age. But when I looked back to his admission, he completed less than 100 meters with 4 seated rests during that 6 minutes. For the patient, it was very encouraging to see the improvement. If I had not counted [the 6MWT] because he stopped [during the test], then I would have had no value to compare it to.” (IP, hospital 5) “Starting out, I actually loved the app because it helped me understand more and see more. Once I started getting comfortable with the testing, then I started to find that it was holding me back a little bit. It got me going and it got me doing…[When] I was more efficient doing the test, then I would look back at different resources to get my scores and my data.” (IP, hospital 7) “I remember this patient who lacked insight into her limitations. When she had to go back [home], it was very hard for her to interpret what we were saying. But when we were doing the test, she would jot down the numbers…over days, this gave her feedback, and she sort of compromised a little bit and understood her condition better. In a way, that helped because she had the numbers to look at…She knew she wasn’t making as much gains as one would require to be a community ambulator.” (IP, hospital 8)

^*a*^6MWT = 6-Minute Walk Test; 10MWT = 10-Meter Walk Test; CMO = context-mechanism-outcome; IP = inpatient; OP = outpatient; PL = professional leader; PPL = professional practice leader; PT = physical therapist; TIA = transient ischemic attack.

^*b*^Organized by domains in the Theoretical Domains Framework.

### CMO1: No Onsite Facilitator? No Practice Change in Acute Care

Participants providing acute stroke care on non-neurology services (eg, palliative/emergency care) at sites without an onsite facilitator lacked authority to facilitate the implementation strategy. For instance, physical therapists did not set up a permanent 6MWT walkway due to inadequate space and felt they lacked authority to request to install wall markings for the walkway. Physical therapists indicated that establishing a walkway nearby with onsite PPL support was essential to implementing the tests and a shorter 6MWT walkway would be more practical.

Physical therapists at 1 site with an absent PPL adapted learning sessions by skipping activities, such as reviewing case scenarios, not relevant to their setting. They did not view the video due to technical difficulties. They practiced walk tests on one another but not with patients. At another hospital, physical therapists attended the first session delivered in-person by the offsite PPL and only 1 of 2 subsequent teleconference sessions due to technology failure. Without onsite facilitation, therapists lacked motivation to participate in learning sessions.

Participants regarded select intervention components positively. For instance, reviewing the guide (particularly the “Top 10” reasons to use the walk tests) increased knowledge and acceptability of walk tests and stroke best practices, hands-on activities during learning sessions enabled skill development, and the app facilitated walk test administration based on standardized protocols and access to reference values without needing paper. Despite gains in knowledge and skill, physical therapists perceived the clinical usefulness of walk tests to be limited. The recommended walk test practices were seen as impractical in acute care because most patients could not walk 10 m or for 6 minutes. Setting goals for walking was lower in priority than more achievable goals related to bed mobility and walking to a chair, and length of stay was often too short to evaluate goal achievement. Documenting baseline values was deemed irrelevant because walk tests would be re-administered on admission to inpatient rehabilitation. Physical therapists were disinclined to discuss walking prognosis with patients so early post stroke. These issues contributed to a lack of motivation to administer walk tests, and physical therapists did not intend to use the walk tests in future.

### CMO2: Onsite Facilitation Fostered Integration of Select Practices in Acute Care

When onsite facilitation occurred in acute care settings, walk test administration and use of reference values for patient education were adopted to a variable degree with high functioning patients. Leaders noted that being onsite enabled them to regularly remind physical therapists to use the iWalk resources and coordinate learning sessions (either themselves or by asking therapists to schedule them). PPLs noted that, compared with an administrative role, being in clinical practice gave them more influence over physical therapists.

Physical therapists expressed a willingness to engage with toolkit materials and set up walkways, and, at 2 sites, they had experience using 1 of the tests. The toolkit was deemed sufficient to enable inexperienced therapists to learn how to incorporate standardized measures into their practice. As in CMO1, therapists adapted sessions by skipping activities (eg, practicing walk tests with one another) when they had prior experience. Therapists consistently adjusted walk test indications to avoid administration in patients who required assistance to walk, had comorbidities (eg, arthritis, COPD) or cognitive impairment, or lacked the ability to walk 6 minutes without stopping. Although praising the depth and breadth of the guide, therapists perceived some limitations: a case scenario describing the acute care setting was lacking, setting goals was impractical due to the short length of stay, and audit and feedback were inappropriate due to the high turnover of non-ambulatory patients who could not perform the walk tests.

Participants described the app as easy to use. One therapist said the “app was probably what inspired me the most to sort of change my practice.” They appreciated having quick access to walk test instructions and prompts to provide standardized encouragement. Limitations to the app included the inability to save patient information and difficulty using the swiping function to count 6MWT lengths. For sites with smartphones on loan, storing them away from the practice area deterred their use and the battery sometimes became depleted.

Having an uninterrupted walkway next to patient treatment areas facilitated use of the 6MWT. At 1 site, a unit adjacent to the treatment area was vacated, permitting 6MWT walkway set-up and uninterrupted administration during the study. Similar to CMO1, therapists at 1 site found it difficult to obtain permission to put walkway markings on the wall. One therapist purchased low-cost pylons and tape for walkway set-up, despite the hospital not covering the cost.

Therapists expressed a preference for the 10MWT over the 6MWT because it required less time and could be completed without interruption, was less onerous for the patient, and a greater number of patients “could do the test.” Some therapists observed that administering the 6MWT would not add any information to what they already knew about their patient’s ambulation level and could discourage patients. Others felt the test would still be useful to monitor change in patients who might need breaks. Some therapists described prioritizing measures such as the Berg Balance Scale^24^ over a walk test, because it yielded more valuable information. Many therapists identified gait quality as more important than walking speed or distance.

Therapists across sites felt that some patients benefitted from performing walk tests and receiving education about their walk test performance. For example, 1 patient who was previously a runner and understood speed and distance appreciated learning results from both tests. Therapists described not sharing normative values with individuals with cognitive deficits or people who might become discouraged by comparing their performance. Some physical therapists noted using the walk tests to make recommendations for mobility progression and gait aids. Physical therapists at another site were already using the 10MWT to varying degrees to monitor change in walking capacity. After provision of the toolkit, they began comparing performance to normative values to judge the magnitude of the deficit and to crosswalk speed to determine if patients were safe for community ambulation. Ultimately, physical therapists at this site described continuing this practice, with the addition of using normative values, and intended to administer the 6MWT in patients who could walk 6 minutes. At the remaining 2 sites, therapists either did not intend to use walk tests or committed to using both tests with high functioning patients in future.

### CMO3: Onsite Facilitation Fostered Integration of Most Practices in Inpatient and Outpatient Rehabilitation

When onsite facilitation occurred in inpatient/outpatient rehabilitation settings, many participants incorporated 1 or both tests to evaluate and monitor walking capacity as well as confirm treatment selection and applied reference values for education and goal setting. PPLs set up walkways, which was facilitated by pre-existing markings on the wall and assistance from a co-operative student. Challenges obtaining permission to put markings on the wall were overcome by placing small squares of tape on the wall to demarcate measurements. At 1 site, therapists shortened the existing 45-m 6MWT walkway to 30 m to match the study protocol. Permanent walkways made it easier to use the walk tests because physical therapists did not have to set up and remove the walkway each time.

Professional (practice) leaders were motivated and enthusiastic. They organized learning sessions as outlined in the guide during lunch or at other times. All sites but 1 completed 3 learning sessions despite challenges such as an influenza outbreak.

Similar to acute care settings, PPLs at some sites adapted the learning sessions by summarizing the material in the guide on reference sheets and presentation slides and offering offsite therapists to join 1 session by teleconference. One of these PPLs overseeing 2 sites that were not using the walk tests before the study observed the facilitator role was more work than anticipated. Therapists noted it was difficult to feel a sense of togetherness with therapists at another site during the teleconference and independently organized the third learning session onsite. One PL decided not to practice walk tests during learning sessions because therapists were considered “advanced,” and some were familiar with the tests. At another site, therapists who were unfamiliar with the tests reported that practicing the walk tests with each other helped them implement the walk tests clinically.

Therapists described the guide as comprehensive; some indicated this also made it time consuming to read and difficult to quickly find material. Therapists expressed strong beliefs about incorporating more evidence-based outcome measures in their practice and appreciated the inclusion of evidence supporting the use of the walk tests throughout the guide and reference values.

Some therapists indicated the guide was better suited to junior clinicians and the goal-setting content did not align with their organizational culture of setting interprofessional, client-centered goals. One PPL who was new to the role described having difficulty with facilitating a learning session during which some therapists expressed strong negative reactions to engaging in role play to practice goal setting.

Therapists described valuing and attempting to use both walk tests with patients who walked independently or required minimal assistance or contact guarding and could follow instructions. At 2 sites, some therapists felt strongly that the 6MWT should only be used with patients who could walk continuously for 6 minutes, and others were comfortable permitting breaks, which was acceptable in the protocol because this enabled them to measure progress and encouraged patients to set goals.

Similar to acute care settings, physical therapists used the 10MWT more frequently than the 6MWT, because it has a shorter walkway that could be set up near the treatment area and was less onerous to administer, requiring less time and equipment. The 10MWT was “better suited” to the ambulatory capacity of their clientele, particularly in slow-stream rehabilitation. In some inpatient settings, therapists noted the 10MWT was more aligned than the 6MWT with the therapy goal of independence at home, but not necessarily in the community. Setting up 6MWT walkways far from treatment areas made it too time-consuming to transport patients with low levels of ambulation to the walkway. Some inpatient and outpatient therapists who routinely administered the 2-minute walk test^8^ added or administered the 6MWT instead. Outpatient therapists used various methods, including the 6MWT, 10MWT, 2-minute walk test, observational gait analysis, and the timed “up and go,”^25^ to evaluate gait. One therapist prioritized the 6MWT over the 10MWT because the task was more challenging and aligned better with community ambulation goals. Other outpatient therapists prioritized the 10MWT because it was less time-consuming to administer in a 30-minute treatment session.

Therapists praised the app for giving access to walk test instructions. For the majority, immediate calculation of results and reference values by the app saved time, increased knowledge, and facilitated patient education and goal setting. Some therapists described using the app as a training tool. Therapists at 1 hospital who were already administering both tests reported that using the app helped them integrate reference values for patient education, goal setting, and document %norm values in the health record.

Therapists described a preference for communicating age/sex norms, community ambulation classification, community distances, and crosswalk speed to set realistic expectations with patients about their ability to walk in the community; dispel inaccurate perceptions in some outpatients that their walking status was worsening; and set goals related to community ambulation (eg, speed to cross the street).

## Discussion

This realist evaluation provides insight to help explain 2 principal findings of the quantitative iWalk evaluation: (1) lower level of use of both walk tests in acute care compared with rehabilitation settings, and (2) greater use of the 10MWT compared with the 6MWT across settings. When faced with a practice change initiative, an onsite facilitator was essential to organize and coordinate the implementation strategy. Although the toolkit provided guidance on how to conduct learning sessions and set-up walkways, a local facilitator was needed to obtain approvals for a permanent walkway, schedule and conduct educational sessions, provide reminders to review iWalk guide modules in advance and complete homework, and adapt suggested procedures to accommodate organizational culture and therapist beliefs. When this facilitation was available in the acute care setting, trialing recommended practices with patients led to intentions to administer tests and use normative values for patient education. Carrying out these intentions was feasible in acute care when there was protected time and access to a walkway near the treatment area. Using the tests to monitor change and set goals, however, was deemed infeasible. In contrast, facilitation in inpatient and outpatient rehabilitation settings led therapists to trial and integrate most of the recommended practices. When facilitation occurred, regardless of setting, the majority of therapists preferentially implemented the 10MWT over the 6MWT because of the greater ease of test set-up, administration, and patient performance of the 10MWT.

### Importance of Onsite Facilitation

Across settings, having an onsite facilitator was vitally important. A recent review^28^ highlighted advantages, including familiarity with practitioner’s needs, schedules, clinical roles, caseloads, current knowledge, and past experiences in their practice setting, of embedding someone in a facilitator role in an organization. Familiarity with practitioner’s skills enabled decisions in the current study to skip practice of those skills during learning sessions, thus saving time. Our results underscore additional advantages of being onsite not fully captured by our initial program theory.^9^ Specifically, daily, personal interaction of the onsite PL or PPL with the therapists provided opportunities for reminders. Also, PPLs could boost therapists’ self-efficacy to perform recommended practices by modeling the desired clinical behaviors and encouraging therapists to integrate them.^29^ Local facilitators also needed skills to address local policies limiting permanent walkway set-up, adapt recommended practices to accommodate clinical culture, patient populations, and negative outcome expectations. Health care organizations need to empower clinical leaders/facilitators to use available tools to change practice locally.^30–35^ In 1 study^30^ with supervisor support involving a partnership between acute care and stroke rehabilitation settings owned by the same organization, once acute care hospital administrators agreed to change the color of wall tiles to mark walkway distances for the 10MWT and 6MWT, inpatient and outpatient settings agreed to follow suit.^30^ Study findings can be used to inform the development of training programs and selection criteria for professional leadership roles.

### Importance of Space and Walkway Locations

Findings strongly emphasized the need for permanent walkways near treatment areas to support walk test implementation across settings. A permanent walkway saves time and promotes reliable walk test administration.^30^ Multiple walkways are required if therapists practice in diverse locations in the same facility. Finding space for a 14-m 10MWT walkway was not an issue, but a universal challenge was finding a 30-m distance for the 6MWT. To overcome this barrier, consideration will be given in the next iteration of the toolkit to recommending use of a 15-m walkway for the 6MWT, given evidence of the reliability and validity of that distance.^34^

### Importance of Practice

Findings emphasized the need to practice test administration and use of reference values with colleagues and patients. Training sessions are a consistent element of knowledge translation interventions aimed at changing assessment practice in rehabilitation contexts.^30^^,^^32^^,^^33^^,^^37^ Participants who were unfamiliar with the walk tests recognized the need to practice administering walk tests with each other to build their skill and self-efficacy prior to attempting them with patients. Many with prior experience, however, skipped practicing the walk tests with each other during learning sessions. Although this may seem reasonable, numerous protocols are available for both walk tests,^38^^,^^39^ and physical therapists across settings held inaccurate beliefs about patient eligibility for the 10MWT and the 6MWT. A gap in the study intervention was the lack of a strategy to evaluate walk test protocol fidelity and address beliefs that may have been associated with experience using other protocols. Incorporating strategies into the toolkit to address these issues may also address the limited application of the walk tests to high-level patients without cognitive deficits or comorbidities observed in this study.

### Applicability in Acute Care

Even with onsite facilitation, patient, hospital, therapist, and toolkit factors hindered implementation of recommended practice in acute care settings. Findings suggest that the focus in acute care should be on test administration for evaluation, patient education, and provision of baseline data for those transitioning to inpatient rehabilitation. The toolkit should be revised by adding a case scenario that contextualizes these recommended practices and a learning session agenda specific to the acute care setting.

This study had limitations. We did not capture the perspectives of some stakeholders, such as patients and managers, target settings without PL/PPL positions, triangulate quantitative and qualitative data, or evaluate the sustainability of practice change. Strengths include the rigorous theory-based approach, triangulation of 2 data sources, and the large size of the study that optimizes the transferability of findings to similar centers.

This realist evaluation offered important insights into why implementation of the 10MWT and 6MWT post stroke varied across settings following provision of a toolkit. Findings underscored contextual factors, such as the presence of an onsite facilitator, and activities, including permanent walkway set-up near treatment areas, and practice of test administration and reference value application with peers and patients, that appeared essential to elicit practice change.

## Supplementary Material

Supplemental_file_1_sample_FG_interview_questions_pzab232

## Presentations

Preliminary results from this study were presented at the Canadian Stroke Congress; September 9–11, 2017, in Montreal, Quebec, Canada, and at the 11th World Stroke Congress, October 17–20, 2018, in Montreal, Quebec, Canada.
