# Supplementary material for: Experiences of Physical Therapists and Professional Leaders With Implementing a Toolkit to Advance Walking Assessment Poststroke: A Realist Evaluation
Source: Phys Ther. 2021 Oct 4;101(12):pzab232. doi: 10.1093/ptj/pzab232 (PMC8715419; doi:10.1093/ptj/pzab232)
Supplement: Supplemental_file_1_sample_FG_interview_questions_pzab232 [file supplemental_file_1_sample_fg_interview_questions_pzab232.pdf]

Supplemental file 1. Sample questions posed during focus groups with physical therapists and interviews with professional leaders and professional practice leaders

**Sample questions for physical therapists**<br>• Tell me about what motivated you to participate in this study<br>• Tell me about your experience using the 10-metre walk test and 6-minute walk test among ambulatory patients post-stroke with or without the iWalkAssess app<br>• Tell me about your experiences using the reference values described in the iWalk guide and app<br>• Can you tell me about your experiences with the learning sessions?<br>• What are your opinions about whether the guide was sufficient to enable you to integrate the walk tests into your everyday clinical practice?
**Sample questions for professional leaders and professional practice leaders**[*]<br>• What are the role and responsibilities of your position?<br>• How would you describe your role in the iWalk study?<br>• Tell me about your experience setting up the walkways and organizing the equipment to perform the 10-metre and 6-minute walk tests as outlined in the iWalk guide.<br>• Tell me about your experience organizing the learning sessions<br>• What are your overall impressions of how each learning session went?<br>• What is your opinion about whether physical therapists changed their practice as a result of the iWalk study?

[*]Professional practice leaders were also asked about their experiences using the toolkit in clinical practice.
